# Concept review of dry powder inhalers: correct interpretation of published data

**DOI:** 10.1186/s40248-015-0033-0

**Published:** 2015-12-02

**Authors:** David Price, Henry Chrystyn

**Affiliations:** Academic Primary Care, Division of Applied Health Sciences, University of Aberdeen, Aberdeen, AB25 2ZD UK; Research in Real Life (RiRL), 2 Changi Business Park Avenue 1, Singapore, 486015 Singapore; Research in Real Life Limited, Cambridge, CB24 3BA UK; Talmedica Ltd, St Crispin House, St Crispin Way, Haslingden, Rossendale BB4 4PW UK

## Abstract

Dry powder inhalers (DPIs) are widely used in the clinical practice for delivering therapeutics to patients with lung diseases, such as chronic obstructive pulmonary disease. An overview of current DPIs available on the market from high resistance to low resistance has been reported in a recent review article. We assessed this concept review article and believe this letter provides important additional information regarding the correct interpretation of the data on low resistance DPIs.

## Correspondence

Dear Editors,

A review by Dal Negro (April’s edition of *Multidisciplinary Respiratory Medicine* Journal) addressed the topic of dry powder inhalers (DPIs) and factors regarding their effectiveness in the delivery of treatment [1]. While the concept of this article in reviewing and discussing the array of different DPI devices available is welcomed, the author’s interpretation of the data leading to the conclusion that low resistance DPIs, unlike those with higher resistance, should not be regarded as optimally performing DPI is questionable. This we believe is due to a different perspective of the published data surrounding this type of DPI which can potentially mislead some readers.

The author describes the main characteristics of DPIs and suggests that the performance of a DPI is only affected by two main driving factors, the inspiratory flow rate of the patient and the turbulence produced inside the device. While the author might be trying to simplify this topic for the reader, this statement is inaccurate and does not truly reflect the reality that numerous factors, as well as the inhalation, can impact the performance of a DPI, all of which have been well documented in the literature (excipients [2], drug substance and formulation [3]).

The authors’ main statement that low resistance DPIs require a higher inspiratory airflow rate and effort is not accurate and can be misleading. The major flaw in the review is that the author has misunderstood that it is the turbulent force, that is generated inside a DPI during inhalation to de-aggregate the formulation, is created by the interaction between the patient’s inspiratory effort and the resistance of the device [4]. For a set inspiratory effort, therefore, the inhalation flows when using a low resistance DPI will be greater than when using a DPI with a higher resistance. For example, using the same patients, a mean peak inspiratory flow (PIF) of 72 L/min was generated with the Breezhaler® device compared with a mean PIF of only 36 L/min with the HandiHaler®, a device of significantly higher airflow resistance [5]. The resultant turbulent force, inside the two devices, will be similar [6]. The other two inhalation related factors that affect the de-aggregation of the dose are the inhaled volume required to empty the dose out of the device, and finally, the length of the inhalation channel within the DPI. It is beyond the scope of this letter to discuss these two factors.

The perception of suboptimal particle disaggregation with low resistance DPIs at lower flow rates does not hold true. In fact data published for glycopyrronium and indacaterol demonstrated there was consistent dose delivery performance from Breezhaler® using airflow rates between 50–100 L/min [5, 7]. Similarly, consistent dose delivery across a range of inspiratory flow profiles has also been shown for a DPI with higher resistance [8].

The author reviews several DPIs (low, mid and high resistance) and states that for the low resistance DPI Breezhaler® device, a PIF rate of 111 L/min is ‘*required’* which is not the case. Published data shows that patients with mild to very severe chronic obstructive pulmonary disease (COPD) were able to generate, on average, a PIF rate of 94.8 L/min (range, 52–133 L/min) [7]. Consistent dose delivery from the Breezhaler® device has been reported within the relevant range of flow rates above, corresponding to a reported minimum PIF of 52 L/min or more achieved by all studied COPD patients [7]. It does not mean that such rates are required to actuate the Breezhaler® device successfully in order to successfully deliver the medication.

The inspiratory efforts required by patients’ needs to be considered only when a minimum inhalation flow through a specific DPI is required for efficient de-aggregation to occur [9]. This is not the case with the low resistance DPI Breezhaler® device, as patients were able to achieve the minimum flow required. But when the resistance of the DPI is higher (for example Handihaler® [5, 10], Turbohaler® [11]), then sufficient inspiratory effort to generate the minimum flow (approximately 30 L/min for both the Handihaler® [12] and Turbohaler® devices [13]) for efficient de-aggregation requires consideration. This may be a problem for those who have more severe disease because their inspiratory effort is reduced and thus they have challenges in generating the required threshold flow rate [4, 11, 14].

To conclude, Dal Negro has not accurately represented the data on low resistance DPIs. We hope this letter addresses that need for balance in the interpretation of the data on DPIs.

## Authors response

From the Author:

Dear Editor,

Thank you for allowing me to reply to this letter.

I have read the Letter to the Editor of Multidisciplinary Respiratory Medicine by H. Chrystyn and D. Price with attention, and concerning my recent review on “Dry Powder Inhalers and the right things to remember: a concept review”. First of all, I would like to amend the Authors’ assumption when they report that the review is stating that the high resistance DPIs should be regarded as the optimally performing devices in terms of their inhalation effectiveness. Actually, in the text of the review is clearly written that “only when the inhalation flow rate and the DPI intrinsic resistance are balanced, the inhalation is optimized”. This condition practically corresponds to that of medium resistance devices (and not of that of high resistance ones), even though also a range of conditions exists among the family of medium resistance DPIs (such as, some DPIs are positioned closer to the lower and some other to the upper limits of the medium resistance regimen – see Table 2 in the review). The meaning of this assertion in the review is quite different from what interpreted and reported by the Authors in their letter, likely mis-understanding the real philosophy of the review itself. Secondly, the review stems from recent and consolidated evidences in the literature which are clearly confirming that the two main factors (even not the unique ones, as specified also in the review, pag. 4) are represented by the patient’s lung performance and the intrinsic resistance of the DPI (see references). On the other hand, the mechanism of dispensing powder from a DPI is a function of the Turbulent Energy, according to the following relationship: √P= Q x R, where Q is the inhalation flow and R is the inhaled resistance. Not by chance, when the flow rate is measured at a pressure drop of 4.0kPa, which corresponds to the pressure drop for DPIs testing, the inspiratory flow rate needed by low resistance DPIs is much higher than that of medium, and obviously even more of that of high resistance DPIs (Al-Showair et al.- Respir Med, 2007). Finally, I would like to emphasize that the review is absolutely independent: actually, differently from the letter, all DPI brands reported are equally mentioned within the review, and no brand was privileged in terms of n. of mentions in the text. In conclusion, I am convinced that the review should be regarded as a convenient tool which contributes to refresh some basic (frequently forgotten) factors crucially modulating the DPIs’ performance. It would also provide physicians with some concepts which could support their choices in clinical practice, but stemming from a more independent basis.

Roberto W. Dal Negro

National Center for Respiratory Pharmacoeconomics and Pharmacoepidemiology - CESFAR, Verona, Italy Research & Clinical Governance, Verona, Italy

## Abbreviations

DPI: dry powder inhaler
PIF: peak inspiratory flow
COPD: chronic obstructive pulmonary disease
